# Historical Account of the Diseases, at Different Times, Included under the Name of Leprosy. From Dr. Adams's "Inquiry into the Laws of Epidemics"

**Published:** 1809-11

**Authors:** 


### *Historical Account of the Diseases, at different Times, included under the Name of Leprosy. From* Dr. ADAMS's *"Inquiry into the Laws of Epidemics."*

IT would be a very curious inquiry for any medical man of leisure to trace the great variety of diseases which have at different times been included under the name of leprosy. Among the Jews it is evident that the term was very general in its application, though at different times it might be appropriated to certain well ascertained appearances. The leprosy described in the Levitical law, was not only a curable disease, but probably curable by the unassisted powers of the constitution. The patient was required to retire from society till he was pronounced by the priest to be clean; after which, and the necessary ablutions, he was re-admitted into the congregation. In another place* I have confirmed the conjectures of an ingenious writer, that this disease was probably the same as the yaws in the West Indies and in Africa. Whether in the present days it ever extends as far North as Egypt, I pretend not to say, but it is highly probable that it can never make any considerable ravages where slavery is unknown; that is, where men are tolerably covered, and their skin not much exposed, or much broken. It rarely appears among the Whites in the West Indies, excepting by accidental inoculation, and it is not with certainty ascertained, whether it is ever conveyed, excepting by actual contact, or the communication by the medium of insects.

There is every reason to believe that the leprosy described in Leviticus, was exterminated from the Israelites before their settlement in Palestine. The only leprosy we hear of in the subsequent parts of Holy Writ was evidently incurable, and considered as such. When King Uzziah was discovered to be a leper, he was confined for the rest of his life in a lone house, and a successor appointed. Naaman, the Assyrian Captain, was recommended by the King his master to the King of Israel, that he might be cured by the miraculous agency of Elisha. The curing of a leper appeared something more than could be expected, even from a prophet so highly favoured; and the King expressed his uneasiness lest the only intention of the Assyrian might be a pretext for war. It is enough to say, that Naaman was cured by miraculous agency, to show that no other means were known.

It is, however, worthy of remark, that the Assyrians were under no apprehension of contagion from the disease; as Naaman constantly attended the King, even in his devotions. But Elisha seemed to entertain a different opinion, and refused the least communication with a leper.

He

* "Morbid Poisons."

He sent his directions without seeing the patient, and when the latter returned cured, but without having undergone the necessary ablutions, he was dismissed with a short reply to his expressions of gratitude, and even to his inquiries concerning a scruple of conscience,—" Go in peace."

The lepers, in the New Testament, were evidently considered incurable; and it appears that they were not permitted to be seen in the towns, but associated together without the walls. Four of these applied to our Lord afar off, with a loud voice; they were cured by miraculous agency, and ordered to present themselves to the priest.

It appears by these circumstances, that after the extermination of one kind of leprosy, the same customs were preserved in a certain degree with regard to another which still remained. The only difference was, that as the patients were never found to recover from this last, which was probably the true elephantiasis, their seclusion was for life.

Elephantiasis is known in all warm climates, and in many considered as contagious. Yet the lepers, or lazars, as they are in some places called, are often suffered to wander without any restraint. Such is the case in the island of Madeira, where these unhappy sufferers are among the common beggars, and often the most hideous are selected to excite charity from new comers. They have indeed a hospital, and a small allowance from the government.

It has been said, that after the expedition of the Crusaders, lepers were so common throughout Christendom, that no less than 15,000 Lazarettos were erected for their reception. By this great caution it is supposed that the disease was exterminated.

To suppose that elephantiasis ever was general in Europe, would be to suppose a change in the human constitution subversve of all physical laws. We know from Celsus, and also from Galen, that the disease, if not unknown altogether in the latitude of Rome, was at least so rare, that those authors were unwilling to describe it. Aretæus, who is supposed to have flourished in Capadocia, is the first writer that entered into any detail in describing elephantiasis; he considered it, however, contagious, which probably prevented so close an intercourse as was necessary to render his description accurate.

When the syphilitic poison first appeared, much inquiry was made in the Italian schools to determine whether that disease disease could be discovered in the writings of the ancients. This produced a more minute examination of all their descriptions of leprosy, and it was now discovered that the leprosy of the Greeks was different from the leprosy of the Arabians. The latter they perceived was the elephantiasis of the Greeks, an incurable disease, and almost, if not entirely, unknown among themselves.

Elephantiasis has never been described by any author as a disease that he had seen in England. Through the politeness of Dr. Baillie, I have been consulted for two cases which he met with in his practice, both from the East Indies. One of these remains at this time in a society to which she is no way injurious, and which contributes to soften the misery of her unhappy fate.

The author of the letter to Mr. Perceval, informs us, that he had been eye witness of the ravages of this disease in Ceylon—yet he remains uninfected. May I add, that in the receptacle for lazars, in the neighbourhood of Funchall, I passed the best part of several days, examining without any injury to myself, the disease in all its stages.

All this, I trust, is enough to show that the disease for which 15,000 Lazarettos are supposed to have been erected in Europe, was not elephantiasis: Lepra græcorum would be too trifling to attract general notice.

At one time I conceived these receptacles must have been for small-pox, and that they had gradually fallen into disuse, as it was found that the disease affected the same person only once during life. But as some of the lepers were obliged to enter with all their goods, moveable and immoveable, it seems to follow that they were to remain for life.

In order to comprehend the nature of these establishments, we must be better acquainted with the history of the order of St. Lazarus, from its first erection. There is some dispute among the writers on this subject, whether this order was the same as that of St. John of Jerusalem. I pretend not to form any decision on a question, which nothing would have induced me to examine but the present "Inquiry." It however appears by the author of "*Histoire des ordres religieuses,*" who maintains a contrary opinion, that the orgin of each was similar, that is, a factory of Italian merchants had erected, at a very early period, a chapel, and afterwards a hospital in Jerusalem. The last, principally for the relief of pilgrims and poor sick, who came to visit the holy sepulchre. When the crusading expedition expedition commenced, this hospital was soon found necessary for the wounded soldiers. By degrees an order of knighthood arose, which was divided into soldiers, ecclesiastics, and sick. The lepers were to be attended by lepers, and, what is very remarkable, the master of the order was to be a leper. This might lead us to suppose, that small-pox was included among the descriptions of leprosy, and that it was found necessary, that those who attended the sick, as well as the master of the order, who was to govern the whole, should not be liable to the disease. These conjectures may appear bold; but let those who think so reflect, that we have no records whatever, to afford us any information concerning the first introduction of small-pox. This reflection, if it does not authorise, at least may be said to invite conjecture.

The Knights rendered great services to the Christian cause, not only in Palestine but in Europe, after their expulsion from the East. In the year 1253 they petitioned the pope to permit them to chuse a sound master, as all the leprous Knights had been destroyed in Syria. The pope not only permitted this, but gave them many other privileges. Clement IV. ordered, under pain of excommunication, that whenever the Knights of St. Lazarus should require it, every prelate should lend his hand to force a leper, with his goods, moveable and immoveable, to be under the care of the order. This may be one way of accounting for their immense riches. Besides this, they received great immunities from the different princes.

In 1490 the pope incorporated this order with the Knights of St. John of Jerusalem; but they remained distinct in France, in which country it appears that the order existed till the revolution, under a vow of taking the charge of wounded and sick soldiers.

What the nature of our establishments in England was it is difficult to say, but almost all the bequests which are preserved in favour of the Burton Lazars, mention *fratribus sanis et leprosis,* and some of them *militibus.* It seems probable, that none were admitted into the community but those who took the vows. Stow has a chapter *of leprose persons and lazar houses.* In this he first refers to the Levitical law, next to a decree in the ecclesiastical court, passed in the year 1200, the second of King John, by which, according to the Lateran council, when so many leprous persons were assembled as might be able to build a church, with a church yard, to themselves, they might erect such a church, provided it should not be injurious to the the old churches, and also that they should be excused paying tythes of their gardens, or increase of cattle.

" I have moreover heard, continues he, that there is a writte in our law, de *leproso amovendo*; and I have read that King Edward the Third, in the twentieth year of his raigne, (about 1337 or 8) gave commandment to the mayor and sheriffes of London, to make proclamation in every ward of the city and suburbs, that all leprose persons inhabiting there, should avoid within fifteen days next, and that no man suffer any such leprose person to abide within his house, under pain to forfeit his said house, and to incur the king's further displeasure. And that they should cause the said lepers to be removed into some out places of the fields, from the haunt or company of sound people: whereupon certain lazar houses, as may be supposed, were then builded without the city, some good distance; to wit, the Lock without Southwarke, in Kent Street; one other betwixt the Miles-End and Stratforde, Bow; one other at Kingsland, betwixt Shoreditch and Stoke Newington; and another at Knightsbridge, west of Charing-Cross. These four I have noted to be erected for the receipt of leprose people sent out of the city at that time."

The only one of these houses now remaining is the Lock, near Hyde Park. Those of Kent Street, and of Kingsland, have been consolidated into St. Bartholomew's Hospital, which has a ward under the name of Lazarus, for the reception of the same objects as the Lock is confined to. Of the fourth, at Mile End, I can learn no particulars. I have heard Mr. Pott speak of the time, when the worst kind of head-cases, as he said they used to be called, were sent to Kingsland. That building still remains, adjoining the turnpike, and is converted into a manufactory, the chapel only being preserved for its original purposes. By all this it would appear, that leprosy was a term used for any severe cutaneous disease, or foul obstinate ulcers, which the ignorance or indolence of medical practitioners might choose to confound under one common name, and which afterwards, for many years, acquired a different appellation. This is confirmed by a passage in Archibald Pitcairn, who expressly tells us, *Lepra autem ante famam morbi Neapolitani hydrargyro cessit*; NOMENQUE NUNC AMISIT.

But these establishments were quite distinct from those which were under the direction of the religious orders, and accordingly, most of the latter were seized by Henry as church lands, whilst the former exist in some form to this day, as charitable institutions.

I have taken much pains to discover the passage in Mathew Paris, in which he mentions that 18000 establishments for lepers were erected in different parts of Christendom. But all my diligence in research as well as enquiries of others have been insufficient. The only thing I have discovered that bears on the question is, that the Templars had 9000 manors, and the Hospitaliers 19000 in Christendom, besides other emoluments; yet, with all this, in the year 1244, they petitioned for assistance by circular letters among the Christians, to enable them to build a fort for the protection of the Holy Land against the Saracens. The Christians, however, calculated that each manor might at least send a single soldier completely equipped to Palæstine. This and the recollection of the perpetual quarrels between the two orders, and their ill behaviour in other respects, determined the Christians not to pay any regard to their application for assistance.

Whether these *manerii* were any thing more than freehold property, however inconsiderable, I leave to be determined by the lawyers; but by a reference to the various bequests and endowments to be found in Mr. Nichols's "Appendix to the Burton Lazars," it will appear, that many of their possessions in land were inconsiderable.

The nature of the disease which constituted a leper, appears to have varied according to the country in which the term was used; Lorry has some good remarks on this subject. After observing how little was known of the true Elephantiasis or Syrian leprosy, he adds, "Auctores qui in regionibus borealibus scripserunt lepræ nomen multis, etiam dederunt morbis cutaneis." The number of lepers he tells us, on the authority of Raimond, was so much increased after the holy war, that 1040 establishments were raised for their reception in France, of which only the names are left, the places being empty. *Imo equestris ordo apud Gallos iis inserviendis natus, ad alios usus postea ob inutilitatem detortus est.*

I leave it to others to unravel all this obscurity. But so much is certain, that elephantiasis never could have been common, if at all known, in England; that in the countries where it is most common, its contagious is much less certain than its hereditary property. That the term leprosy was given to most cutaneous diseases not otherwise designated, and that all nations are more infested with such diseases in proportion as they are poorer, or have less inducements to cleanliness.

The only instance I have met with of any description of of the disease, in a legal sense, is in a passage of Rymer's Fœdera, referred to by the learned judge. In this it appears, that Joanna Nightengale, of Brentford, was accused by some of her *good-natured* neighbours of being a leper. A writ was issued, by which the king's physicians were to examine her. In this case there was only one mode of proceeding, namely, to search for authorities; and by these they found, that Joanna did not come within the description of such as had been proscribed in other countries. If any authority could be produced of a person coming under the description required by this decision, it must then be allowed that elephantiasis has been known in England. On the whole, it appears evident that the term leprosy has been much too general, and perhaps is so at this time, even among medical writers. This would have been less inconvenient, had it been always used only in a generic sense; but by considering it as a specific disease, many appearances arising from different causes have been confounded, and words which should express distinct complaints, have been used as mere synonyms.

It is hardly credible, how little Lexicographers are aware of the importance of words. It cannot be necessary to observe, that the term *Lazaretto* is Italian. In another place* I have remarked, that the *Della Crusca* Society has set many of the philosophers in Europe at variance, by their arbitrary explanation of the word *pellicello*. A similar inattention seems to have had its effect in the word Lazaretto. Antonini, who professes to follow that learned Society, translates the word as it is now used in England and France; that is, a deposit for men or goods suspected of conveying the plague. He gives the Latin word, *Læmocomium*; whether coined at the same mint I pretend not to determine, but in an Italian and English Dictionary, published in London in the year 1611, the word Lazaretto is translated an *Hospital for sick folks.* Of this kind, it is most probable, were many of the Lazar houses on the continent, or rather the houses under the religious order of St. Lazarus; and the union of that order with the Hospitaliers, is a further confirmation of the same. If these remarks appear bold, the reader will make some allowance for the trouble it costs to reconcile authorities, by which we expect only to be instructed. In a few years an old dictionary may be worth its weight in gold.

* In a Paper on the Acarus Scabiei, read before the Royal Society, and published in "Morbid Poisons."

CRITICAL

